# MtlD as a therapeutic target for intestinal and systemic bacterial infections

**DOI:** 10.1128/jb.00480-24

**Published:** 2024-12-27

**Authors:** Andrew Schwieters, Allysa L. Cole, Emily Rego, Chengyu Gao, Razieh Kebriaei, Vicki H. Wysocki, John S. Gunn, Brian M. M. Ahmer

**Affiliations:** 1 Department of Microbiology, The Ohio State University 142712 , Columbus, Ohio, USA; 2 Infectious Diseases Institute, The Ohio State University 142712 , Columbus, Ohio, USA; 3 Center for Microbial Pathogenesis, Research Institute at Nationwide Children's Hospital 51711 , Columbus, Ohio, USA; 4 Department of Veterinary Biosciences, The Ohio State University 142712 , Columbus, Ohio, USA; 5 Campus Chemical Instrument Center, The Ohio State University 142712 , Columbus, Ohio, USA; 6 Department of Outcomes and Translational Sciences, The Ohio State University 142712 , Columbus, Ohio, USA; 7 Department of Chemistry and Biochemistry, The Ohio State University 142712 , Columbus, Ohio, USA; 8 National Resource for Native MS-Guided Structural Biology, Columbus, Ohio, USA; 9 Department of Pediatrics, The Ohio State University College of Medicine 2647 , Columbus, Ohio, USA; 10 Department of Microbial Infection and Immunity, The Ohio State University 142712 , Columbus, Ohio, USA; University of Virginia School of Medicine, Charlottesville, Virginia, USA

**Keywords:** *Salmonella*, mannitol, sugar-phosphate toxicity, MtlD, *Escherichia coli*, *Cronobacter sakazakii*, *Pseudomonas aeruginosa*

## Abstract

The ability to treat infections is threatened by the rapid emergence of antibiotic resistance among pathogenic microbes. Therefore, new antimicrobials are needed. Here we evaluate mannitol-1-phosphate 5-dehydrogenase (MtlD) as a potential new drug target. In many bacteria, mannitol is transported into the cell and phosphorylated by MtlA, the EIICBA component of a phosphoenolpyruvate-dependent sugar phosphotransferase system. MtlD catalyzes the conversion of mannitol-1-phosphate (Mtl-1P) to fructose-6-phosphate, which enters the glycolytic pathway. Mutants lacking *mtlD* are sensitive to mannitol due to accumulation of Mtl-1P. Here, we constructed *mtlD* mutants in four different bacterial species (*Cronobacter sakazakii*, *Pseudomonas aeruginosa,* five serovars of *Salmonella enterica*, and three strains of *Escherichia coli*), confirming and quantifying their mannitol sensitivity. The quantification of mannitol sensitivity *in vitro* was complicated by an inoculum effect and a resumption of growth following mannitol intoxication. The rate of resumption at different mannitol concentrations and cell population densities is fairly constant and reveals what is likely an intoxication processing rate. Provision of mannitol in drinking water, or by intraperitoneal injection, dramatically attenuates infection of a *Salmonella enterica* serovar Typhimurium *mtlD* mutant in mouse models of both gastroenteritis and systemic infection. Using CC003/Unc mice, we find that a *mtlD* mutant of *Salmonella enterica* serovar Typhi is also attenuated by provision of mannitol in drinking water. Therefore, we postulate that MtlD could be a valuable new therapeutic target.

**IMPORTANCE:**

The ability to treat infections is threatened by the rapid emergence of antibiotic resistance. Mannitol is a polyol used in human medicine and the food industry. During catabolism of mannitol, many bacteria transport mannitol across the inner membrane forming the toxic intermediate mannitol-1-phosphate (Mtl-1P). Mtl-1P must be processed by mannitol dehydrogenase (MtlD) or it accumulates intracellularly, causing growth attenuation. We test and confirm here that *mtlD* mutants of *Escherichia coli* (including UPEC, and EHEC), *Salmonella* (including serovars Typhi, and Paratyphi A, B, and C), *Cronobacter*, and *Pseudomonas* experience mannitol sensitivity *in vitro*. Furthermore, providing mannitol in drinking water can alleviate both gastrointestinal and systemic *Salmonella* infections in mice. This suggests that inhibition of MtlD could be a viable antimicrobial strategy.

## INTRODUCTION

Bacterial infections are becoming harder to treat due to the growing prevalence of antibiotic resistance, motivating the need to identify new drug targets. We highlight here the difficulties in treating *Salmonella enterica*, a Gram-negative bacterial species comprising over 2,500 serovars that cause diseases ranging from gastroenteritis to typhoid fever. Nontyphoidal serovars like Typhimurium typically cause self-limiting gastroenteritis with severe inflammation lasting 4–10 days (1) and shedding of bacteria continuing for up to 5 weeks (2). Serovar Typhimurium induces inflammation in the gut using two type 3 secretion systems, T3SS1 and T3SS2, encoded on separate pathogenicity islands (SPI1 and SPI2, respectively) (3–5). Inflammation is advantageous for the pathogen as it eliminates competing microbial species and generates respiratory electron acceptors such as nitrate (6) and tetrathionate (7) (reviewed in [5, 8, 9]). Paradoxically, antibiotic treatment can prolong shedding and worsen outcomes, presumably due to the depletion of the protective host microbiota (10–17). Therefore, antibiotic use is typically reserved for those with severe illness or at risk for invasive disease, with treatment focusing instead on hydration therapy to replace lost water and salts (18).

Strains of nontyphoidal serovars are evolving rapidly to cause invasive systemic disease in Africa, where invasive disease is coincident with malaria, sickle cell disease, and AIDS (19, 20). Two sequence types (ST) currently predominate in infections leading to salmonellosis: serovar Typhimurium ST313 and serovar Enteritidis ST11 (21). These invasive nontyphoidal *Salmonella* (iNTS) are now the most common cause of bacteremia in Africa, and they have a high prevalence of multiple drug resistance (>47% of isolates) (22, 23). The typhoidal serovars (Typhi, Paratyphi A, and a few others) are adapted to humans and cause typhoid (or enteric) fever. The case fatality rate was 10-20% prior to the discovery of antibiotics, which subsequently reduced mortality to 1% primarily through the use of chloramphenicol (24). Between 2% and 5% of people infected with serovar Typhi become chronic carriers that shed the bacterium in their stool for years, a condition recalcitrant to chloramphenicol treatment (24–27). Multi-drug-resistant (and recently, extensively drug resistant) strains of serovar Typhi have emerged and are spreading rapidly (28). These strains are particularly concerning as they are resistant to the once-successful fluoroquinolones (e.g., ciprofloxacin) and third-generation cephalosporins (29). Every year there are more than 10 million cases of typhoid fever that result in 100,000 deaths (30, 31). The Centers for Disease Control and Prevention and the World Health Organization have listed both the typhoidal and nontyphoidal *Salmonella* serovars as a threat because multiple-drug resistance is prevalent and increasing among these organisms (32, 33).

**FIG 1:**
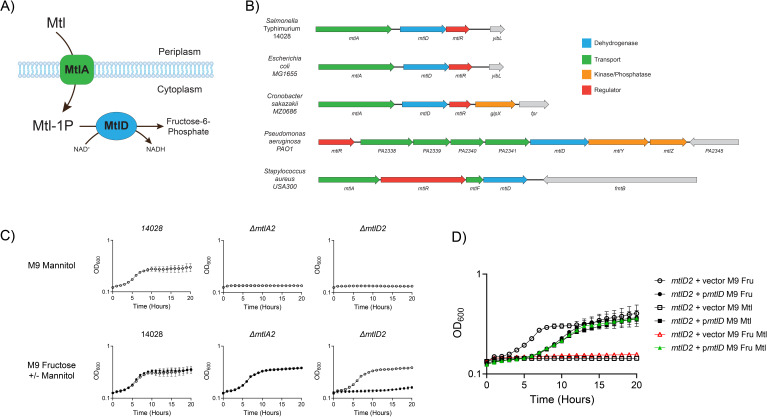
Mannitol catabolism and intoxication. (**A**) Schematic of mannitol catabolism in *E. coli* and *Salmonella*. Periplasmic mannitol is imported into the cytoplasm by MtlA (in green, the EIICBA component of a phosphoenolpyruvate-dependent sugar phosphotransferase system), producing mannitol-1-phosphate (Mtl-1P). Mtl-1P is oxidized to fructose-6-phosphate by d-mannitol-1-phosphate 5-dehydrogenase (MtlD, blue), generating NADH from NAD^+^. (**B**) Mannitol utilization loci in *Salmonella enterica* serovar Typhimurium (14028), *Pseudomonas aeruginosa* (PAO1), *Staphylococcus aureus* (USA300), *Escherichia coli* (MG1655), and *Cronobacter sakazakii* (MZ0686). (**C**) Growth of *Salmonella* wild-type (14028), Δ*mtlA2* (AMS300), and Δ*mtlD2* (AMS302) in M9 + 5 mM mannitol (top row), or M9 + 5 mM fructose ± 1 mM mannitol (bottom row). In the bottom row, open symbols are M9 fructose and closed symbols are M9 fructose mannitol. (**D**) Plasmid complementation of Δ*mtlD2* mutation restores function. Growth of Δ*mtlD2* mutant carrying plasmid-encoded *mtlD* (pAMS394) or vector (pWSK29) in M9 + 5 mM fructose, M9 + 5 mM mannitol, or M9 + 5 mM fructose and 1 mM mannitol.

Sugar-phosphate toxicity is a phenomenon in which the blockade of a sugar utilization pathway, either with a mutation or an inhibitor, leads to the accumulation of a toxic phosphorylated intermediate that attenuates growth (34). These toxicities were first observed in the late 1950s during the initial discoveries of sugar utilization pathways in *Escherichia coli* and *Salmonella* (35–40). The phenotypic defects suffered by mutants that accumulate a toxic intermediate can vary and include both bacteriostatic and bactericidal outcomes (34). Sugar-sensitive mutants are inhibited by the presence of the sugar (e.g., *mtlD* in Fig. 1C) as opposed to those that simply cannot utilize the sugar, which are referred to as sugar negative (e.g., *mtlA* in Fig. 1C). The mechanisms underlying sugar-phosphate toxicity remain largely unknown (reviewed in (34)) and their induction as a therapeutic strategy has not been widely explored (41).

Mannitol is a sugar alcohol widely present in nature and synthesized by plants and fungi for use in osmotic regulation and redox protection. Mannitol is metabolically inert in humans (42). In *Salmonella* and *E. coli*, mannitol is catabolized by two gene products: MtlA and MtlD (Fig. 1A). MtlA is the EIICBA component of a phosphoenolpyruvate-dependent sugar phosphotransferase system (PTS) that imports and phosphorylates d-mannitol (hereafter, mannitol), forming mannitol-1-phosphate (Mtl-1P) (43, 44). Mtl-1P is converted to fructose-6-phosphate by mannitol-1-phosphate 5-dehydrogenase (MtlD, M1PDH) (45, 46). The *Pseudomonas aeruginosa* system utilizes an ABC transporter and an intracellular kinase to transport and phosphorylate mannitol, rather than a PTS (47) (Fig. 1B). The sensitivity of *mtlD* mutants to mannitol has been previously demonstrated in *Salmonella enterica* serovar Typhimurium, *E. coli*, and *S. aureus* (41, 48–51). In serovar Typhimurium and *E. coli*, toxicity appears to be bacteriostatic *in vitro*, though lysis has been reported in some mutants (52).

MtlD is encoded by many pathogenic species, suggesting it may be a suitable drug target against a variety of infectious bacteria (53). However, there is limited information on the conservation of mannitol sensitivity and the degree of attenuation of *mtlD* mutants *in vivo*. Here, we report on mutants of *mtlA* and *mtlD* in serovars of *Salmonella* representing typhoidal, nontyphoidal, and invasive nontyphoidal (iNTS) lineages. Mutations of *mtlA* and *mtlD* were also constructed in enterohemorrhagic and uropathogenic *E. coli* (EHEC and UPEC, respectively), *Cronobacter sakazakii*, and *P. aeruginosa*. We find that all *mtlD* mutants are sensitive to mannitol and all *mtlA* mutants are unable to catabolize mannitol. We discovered that quantifying mannitol sensitivity is complicated by both an inoculum effect and the ability of *mtlD* mutants to recover from intoxication.

We previously found that serovar Typhimurium *mtlD* mutants are highly attenuated in the gastrointestinal tract of streptomycin-treated mice while in competition against their wild-type counterpart (53). However, the presence of the wild-type precluded the measurement of inflammation caused by the mutant. By infecting mice with only the *mtlD* mutant, we find that the provision of mannitol in the drinking water can drastically reduce both burden and inflammation. Next, we examined the potential of mannitol to treat systemic infections and found that mannitol provided either intraperitoneal (IP) or in drinking water attenuates a *mtlD* mutant in the spleen and liver. Providing mannitol in drinking water also enhanced the survival of mice infected with the *mtlD* mutant. Finally, an experiment using a recently described typhoidal mouse model (54) suggests that mannitol can attenuate systemic infections by a *mtlD* mutant of serovar Typhi. In conclusion, we find that mannitol sensitivity is conserved among *mtlD* mutants, and providing mannitol to infected hosts attenuates infections in the gastrointestinal tract, spleen, and liver.

## RESULTS

### *mtlD* mutants of four species are mannitol sensitive while *mtlA* mutants are mannitol negative

In the 1970s, it was reported that *mtlD* mutants of *Salmonella enterica* serovar Typhimurium are sensitive to mannitol (48, 52), and we recently confirmed this with serovar Typhimurium strain 14028 (53). It should be noted that mutation of *mtlD* by insertion of antibiotic resistance genes can influence mannitol toxicity phenotypes, likely by altering the expression of the downstream regulatory gene, *mtlR* (Fig. 1B) (53, 55, 56). We previously characterized a nonpolar Δ*mtlD1* mutation that was constructed using lambda-Red recombination followed by FLP-mediated excision of an antibiotic resistance marker (53). Here, we constructed a second nonpolar, in-frame, deletion of *mtlD* using allelic exchange (Δ*mtlD2*) as well as a Δ*mtlA2* mutation (strains and plasmids are listed in Table S1). As discussed below, the phenotypes of the Δ*mtlD1* and Δ*mtlD2* alleles are indistinguishable. Mutations of this type were then made in several other strains (Table S1). For *Salmonella*, these included serovar Typhimurium strain ST4/74, the iNTS strain D23580 (ST313 lineage 2), serovar Typhi strain Ty2, and three paratyphoid serovars (A, B, and C). We constructed *mtlA* and *mtlD* mutations in *E. coli* K12 strain MG1655 and two pathogenic *E. coli* strains (enterohemorrhagic strain 700927 [EHEC] and uropathogenic strain UTI89 [UPEC]). We also constructed mutants of *C. sakazakii* MZ0686 lacking *mtlA* or *mtlD*, as well as a mutant of *P. aeruginosa* PAO1 lacking *mtlD*. Along with the phenotyping and quantification of mannitol sensitivity below, we complemented the 14028 Δ*mtlD2* mutation with *mtlD* expressed from a plasmid (Fig. 1D). While plasmid-based expression of *mtlD* caused a delay in growth, it also restored growth on mannitol as a sole carbon source and eliminated the sensitivity phenotype, confirming that mannitol sensitivity is due to the loss of *mtlD*. In Fig. S1, we quantitated CFU during growth in the presence of mannitol and determined that (1) OD_600_ correlates with CFU, and (2) that mannitol is bacteriostatic to the 14028 Δ*mtlD2* mutant.

Each wild-type strain and its isogenic *mtlA* and *mtlD* mutant were assayed for *in vitro* growth phenotypes (Fig. S2). When grown in rich media (Lysogeny Broth [LB]) either with or without mannitol, *mtlA* mutants grew comparably to wild-type. In the presence of mannitol, all *mtlD* mutants have growth defects by late exponential phase. In some cases (e.g., 14028, Paratyphi B), *mtlD* mutants have subtle growth defects in LB that occur during mid-exponential phase of growth and resolve by stationary phase. This growth defect may be due to the presence of mannitol in LB broth, which we measured at 67 ± 2.3 µM (see “Materials and Methods”). In some cases (e.g., 14028, ST4/74), *mtlD* mutants grown in LB with 5 mM mannitol have decreases in OD_600_ during the stationary phase. This decrease may be from lysis of intoxicated cells, as was previously observed in nutrient broth supplemented with mannitol (52).

Each strain was also assayed in a defined minimal medium (M9) containing either glucose, fructose, or mannitol as a sole carbon source (Fig. S2). For auxotrophic strains, the media were supplemented with casamino acids and tryptophan (referred to as M9 Supp). The presence of mannitol in cultures growing on fructose inhibited the growth of all *mtlD* mutants (but not *mtlA* mutants), indicating that they are mannitol sensitive. Growth inhibition of the *mtlD* mutants is not as severe when glucose is used as the primary carbon source, likely due to catabolite repression (57, 58). When the strains are grown with mannitol as the sole carbon source, neither *mtlA* nor *mtlD* strains can grow, indicating they are mannitol negative and that both genes are essential for mannitol utilization (Fig. 1C; Fig. S2). For strains grown in M9 Supp, mannitol increases growth of wild-type but not *mtlA* mutants.

While each strain background behaved similarly, there are four exceptions worth noting. First, the EHEC *mtlD* mutant has a partial growth defect in M9 fructose (but not M9 glucose). Second, the *mtlA* mutant of serovar Paratyphi A grows in M9 Supp with no primary carbon source added but fails to grow in M9 Supp containing 5 mM mannitol. Third, wild-type UPEC strain UTI89, but not its *mtlA* mutant, exhibits apparent sensitivity to mannitol at high concentrations (5 mM) and lyses beginning 5 h into growth. This sensitivity in the wild-type strain is unlike that seen in *mtlD* mutants which are unable to grow at all. Interestingly, the UTI89 *mtlD* mutant exhibits a similar drop in OD_600_ after recovering from intoxication (Fig. S3). Finally, *P. aeruginosa mtlD* mutants (both our constructed strain and a transposon mutant from the Manoil collection) (59) lack significant mannitol sensitivity when grown in LB or M9 glucose but display typical mannitol sensitivity in M9 fructose. These four observations were not investigated further in this study. In conclusion, *mtlA* is essential for mannitol catabolism and mutation of *mtlD* confers sensitivity to mannitol in all strains tested.

### The serovar Typhimurium *mtlD* mutant is attenuated in C57BL/6 mice at systemic infection sites when mannitol is provided

**FIG 2:**
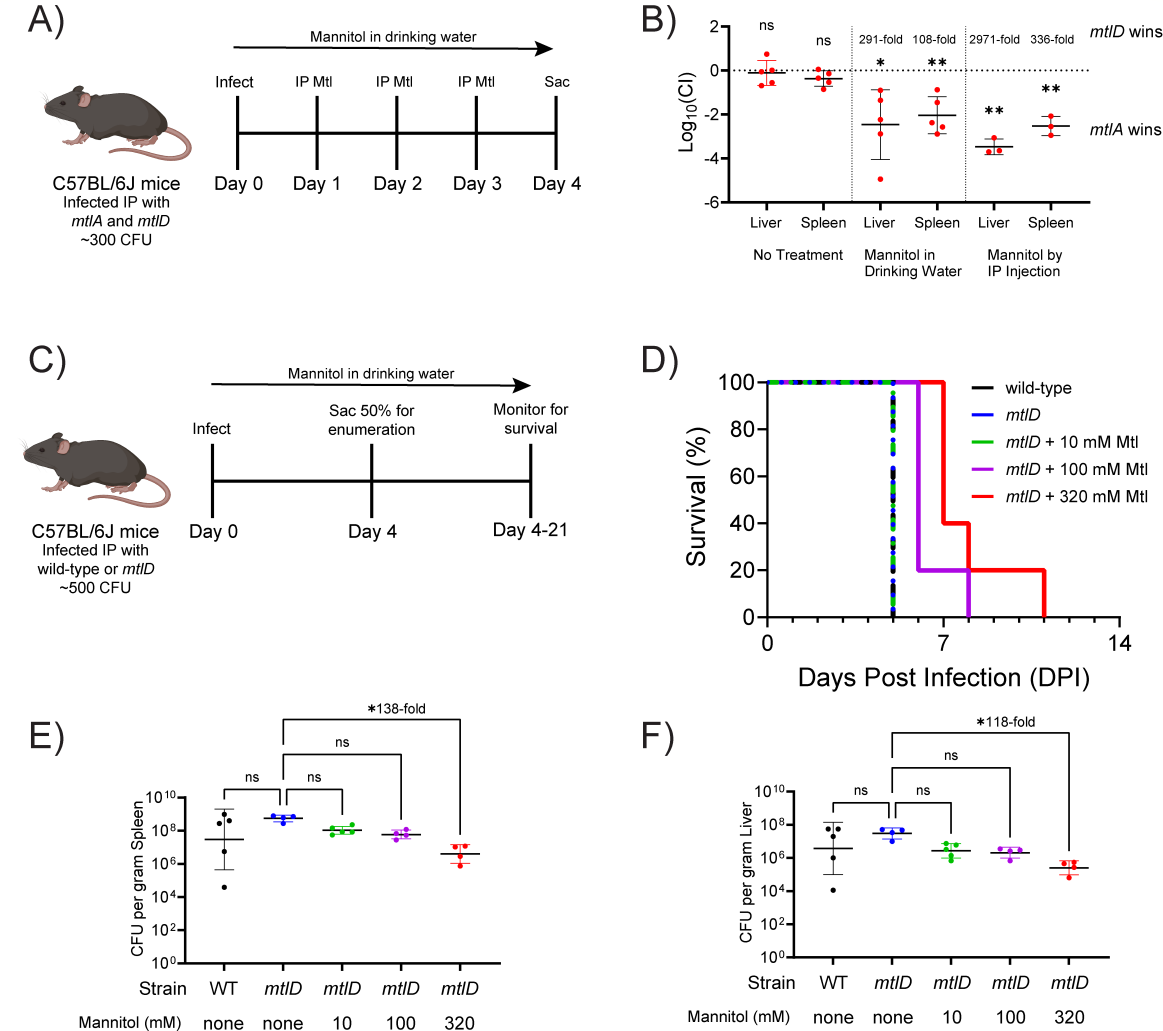
A *Salmonella enterica* serovar Typhimurium *mtlD* mutant is attenuated during systemic infection of C57BL/6 mice when mannitol is provided. (**A, B**) Groups of five C57BL/6 mice were inoculated with a 1:1 ratio of *mtlA1::cam* (EFB036) and Δ*mtlD1* mutant (AMS276) by the IP route, totaling 300 CFU. One group was provided mannitol (100 mM) in their drinking water immediately after infection for the duration of the experiment. Another group was provided mannitol (100 µL of 1 M, equivalent to ~1 g/kg) by the IP route on days 1, 2, and 3 post-infection. A third group received no mannitol. (**B**) On day 4, the burden of Δ*mtlA1::cam* and Δ*mtlD1* mutant serovar Typhimurium in the spleen and liver was determined by dilution plating on LB cam (*mtlA*) and LB kan (*mtlD*) to distinguish the two strains. The competitive index is plotted, calculated as log_10_ of the *mtlA* to *mtlD* ratio, normalized to the initial ratio (0.8:1). Statistical significance was evaluated using a one-sample, two-tailed t-test. *P < 0.05, **P < 0.01. (**C–F**) Groups of 10 C57BL/6 mice were inoculated with 500 CFU of wild-type (14028) or Δ*mtlD2* mutant (AMS302) serovar Typhimurium by the IP route. Mannitol was provided in drinking water immediately after infection at either 0, 10, 100, or 320 mM for the duration of the experiment. (**D**) Kaplan-Meier plot of survival. The log-rank (Mantel-Cox) test and Gehan-Breslow-Wilcoxon test both indicate that the 100 mM and 320 mM groups are different than the other three groups (*P* < 0.01). The Gehan-Breslow-Wilcoxon test, but not the Mantel-Cox test, indicates that the 320 mM group is different than the 100 mM group (*P* < 0.05). On day 4, 5 mice from each group of 10 were sacrificed for enumeration of bacterial burden in the spleen (**E**) and liver (**F**). Fold differences in burden are indicated and statistical significance was evaluated using Tukey’s multiple comparison test. *P < 0.05.

The extraintestinal sites of infection are of major importance in *Salmonella* pathogenesis, particularly for the typhoidal and iNTS lineages. It has been previously noted that a *mtlD* mutant of *Staphylococcus aureus* is attenuated in the liver of C57BL/6 mice (41). To determine if *Salmonella mtlD* mutants are attenuated during systemic infection, a competition experiment was performed. C57BL/6 mice were infected IP with 14028 *mtlA* and *mtlD* mutants together in a 1:1 ratio. The *mtlA* mutant was used instead of wild-type to avoid the wild-type gaining an advantage from utilizing mannitol as a carbon source. Mice were then treated with mannitol by two routes, IP and through drinking water. In human medicine, IV bags are used that contain between 5% and 25% mannitol w/v (274 mM to 1.37 M), with 20% mannitol being the most common. A human dose ranges between 0.5 and 2.0 g/kg; 1.0 g/kg was used here (Fig. 2A). After 4 days, mice were euthanized, and the number of each mutant present in the spleen and liver was determined by plating homogenized organs (Fig. 2B). The *mtlA* and *mtlD* mutants had equal fitness in the absence of mannitol treatment. Providing mannitol in either drinking water or by IP injection led to the *mtlA* mutant outnumbering the *mtlD* mutant in both the liver and spleen by >100-fold. These results indicate that mannitol is accessible to *Salmonella* in both the liver and spleen and that inactivation of *mtlD* confers significant, mannitol-dependent defects during systemic infections.

To determine whether Mtl-1P intoxication could promote survival of infected mice, we performed infections in which each mouse was infected with only one strain. C57BL/6 mice were infected IP to initiate systemic infection and then treated with mannitol in drinking water at three different concentrations (10 mM, 100 mM, or 320 mM), or not treated (Fig. 2C). We arrived at the 320 mM concentration by using as reference the typical concentration of sugar in a can of soda. On day 4, half of the mice were euthanized for enumeration of bacterial burden in the spleen and liver. The remaining mice were tracked for survival. The highest dose was required to significantly reduce bacterial burden in the liver and spleen (Fig. 2E and F). Both 100 mM and 320 mM mannitol treatments delayed mortality compared to the other groups (Fig. 2D).

**FIG 3:**
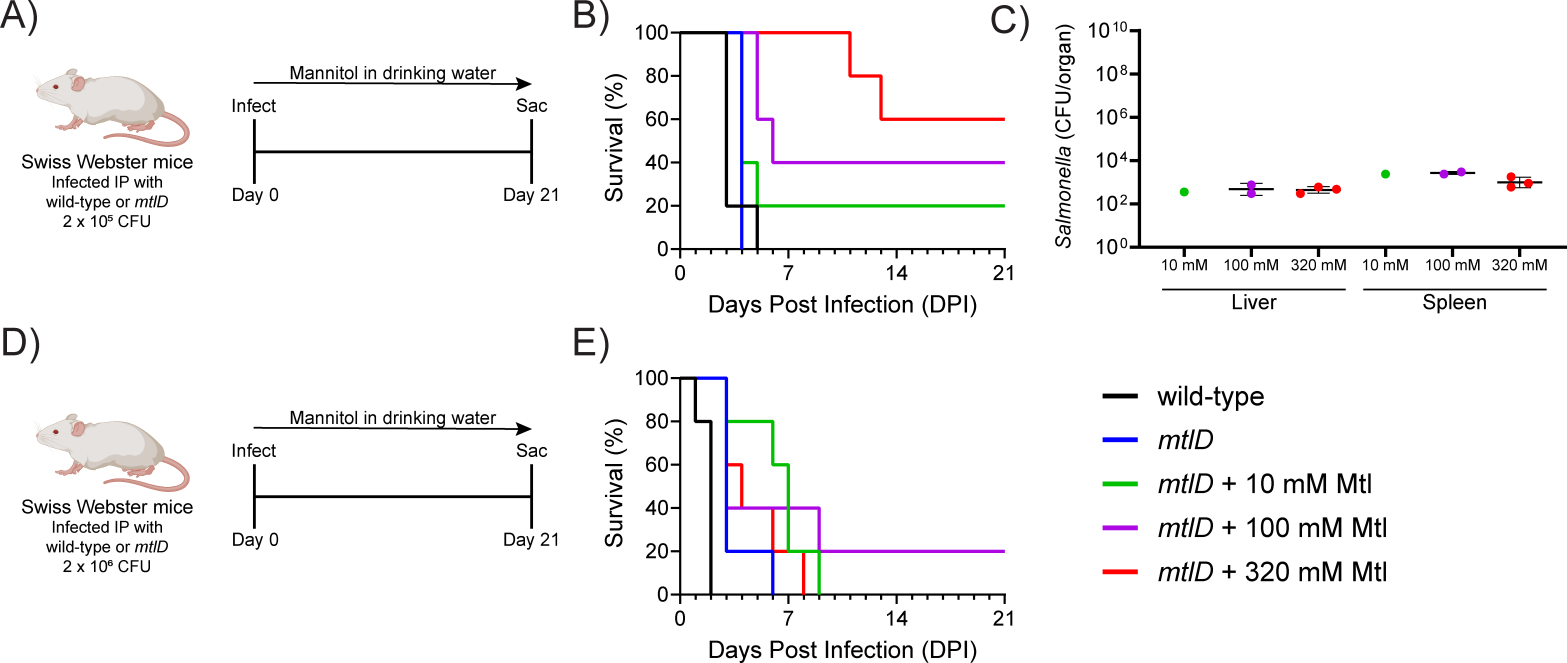
A *Salmonella enterica* serovar Typhimurium *mtlD* mutant is attenuated during systemic infection of Swiss Webster mice. Groups of five Swiss Webster mice were inoculated IP with 2 × 10^5^ CFU (**A–C**) or 2 × 10^6^ CFU (**D, E**) of wild-type serovar Typhimurium (14028) or Δ*mtlD2* mutant (AMS302). Mannitol was provided in drinking water immediately after infection at either 0, 10, 100, or 320 mM for the duration of the experiment. Survival was monitored over 21 days (**B, E**). On day 21, surviving mice from panel (B) were sacrificed for enumeration of bacterial burden in the spleen and liver (**C**). **(B**) The Gehan-Breslow-Wilcoxon test indicates that the 320 mM group is different than the wild-type and *mtlD* groups (*P* < 0.01) and the 10 mM group (*P* < 0.05) but not the 100 mM group. The log-rank (Mantel-Cox) test indicates that the 320 mM group is different than the wild-type and *mtlD* groups (*P* < 0.01) but not the 10 mM or 100 mM groups. **E**) The log-rank (Mantel-Cox) test and Gehan-Breslow-Wilcoxon test both indicate that the wild-type group is different than the other four groups (*P* < 0.01).

The survival experiment was repeated using Swiss Webster mice (Fig. 3A). The untreated groups infected with either wild-type or *mtlD* were unable to survive past day 5. In the treatment groups, survival rates increased in a dose-dependent manner: 20% at 10 mM, 40% at 100 mM, and 60% at 320 mM mannitol (Fig. 3B). In the surviving mice, there were some residual bacteria in the spleen and liver at 21 days post-infection (Fig. 3C). In a repeat of this experiment at a 10-fold higher infectious dose, treatment significantly prolonged survival, but only one treated mouse reached the end of the study (Fig. 3D and E). Treatment with mannitol had no apparent adverse effects on the surviving animals, even after 21 days. Swiss Webster mice have an intact *Nramp1* (*SLC11A1*) gene and are more resistant to systemic infection than C57BL/6J mice, which may explain their differences in survival rates (60–62). In conclusion, mannitol in drinking water can reduce bacterial burden, prolong survival, and reduce mortality in mice-infected IP with a serovar Typhimurium *mtlD* mutant.

### Mannitol in drinking water can prevent serovar Typhimurium *mtlD* mutant expansion and inflammation in the gastrointestinal tract

In a previous study, we determined that serovar Typhimurium *mtlD* mutants are highly attenuated in the gastrointestinal tract of streptomycin-treated Swiss Webster mice during competitive infection against the wild-type (53). This attenuation was largely independent of the presence of mannitol in the drinking water (53). The lack of mannitol dependence could be due to the *mtlD* mutant having additional defects beyond mannitol sensitivity or due to the presence of mannitol in mouse chow, which we determined to be 1.6 ± 0.057 mM (see “Materials and Methods”). An unresolved question in this competition experiment was whether the *mtlD* mutant can cause inflammation of the gastrointestinal tract (53).

**FIG 4:**
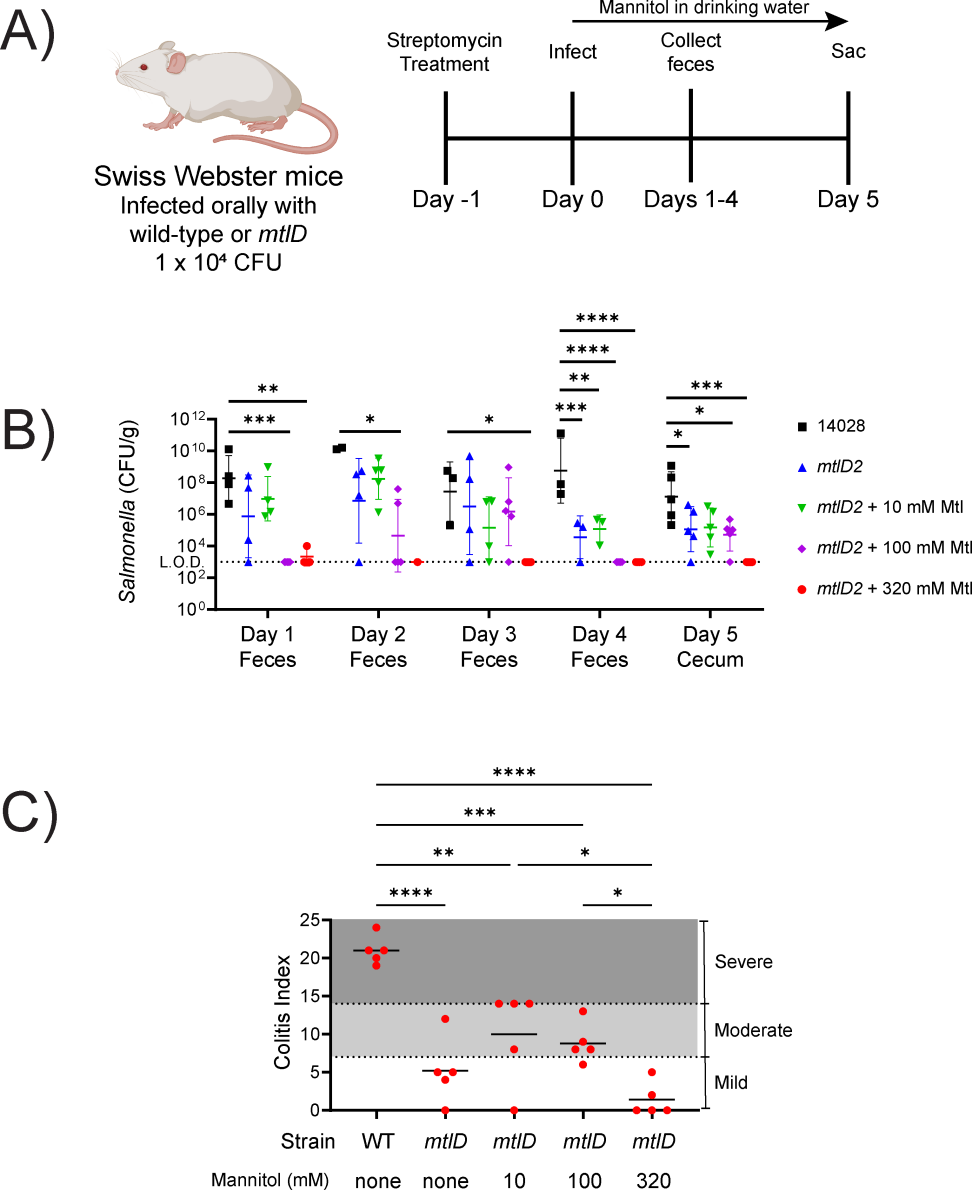
A *Salmonella enterica* serovar Typhimurium *mtlD* mutant is attenuated during gastrointestinal infection of streptomycin-treated Swiss Webster mice. One day after streptomycin treatment, groups of five mice were inoculated orally with 1 × 10^4^ CFU of wild-type serovar Typhimurium (14028) or Δ*mtlD2* mutant (AMS302). Mannitol was provided in drinking water immediately after infection at either 0, 10, 100, or 320 mM for the duration of the experiment. (**B**) Fecal samples were collected daily for enumeration of CFU. On day 5 post-infection, mice were sacrificed for enumeration of CFU in the cecum. (**C**) Histopathological analysis was performed on the proximal colon. Statistical significance was evaluated using Dunnett’s multiple comparisons test (**B**) or Tukey’s multiple comparison test (**C**). *P < 0.05, **P < 0.01, ***P < 0.001, ****P < 0.0001.

To answer this question, we inoculated mice with either the wild-type or *mtlD* mutant alone. The Swiss Webster mice were pretreated with streptomycin, which disrupts the microbiota and renders them susceptible to *Salmonella*-mediated inflammation (17). One day later, they were infected with serovar Typhimurium by oral gavage (Fig. 4A). Mannitol was provided in the drinking water. Feces were collected daily and mice were euthanized on day 5 for enumeration of bacteria in the cecum and for histopathology of the proximal colon. As expected, the mice infected with wild-type *Salmonella* had high bacterial counts and severe inflammation (Fig. 4B and C). The *mtlD* mutant burden in mice treated with 320 mM mannitol dropped below the detection limit (1 CFU/mg) by day 2 and was not detected in the cecum on day 5. All treatment groups (and the untreated *mtlD* mutant) had significantly lower inflammation than the wild-type, dramatically so in the 320 mM mannitol group (Fig. 4C). In conclusion, mannitol can inhibit a *Salmonella mtlD* mutant in the gut and prevent inflammation.

### *In vivo* efficacy of mannitol treatment against a *mtlD* mutant of *S.* Typhi

**FIG 5:**
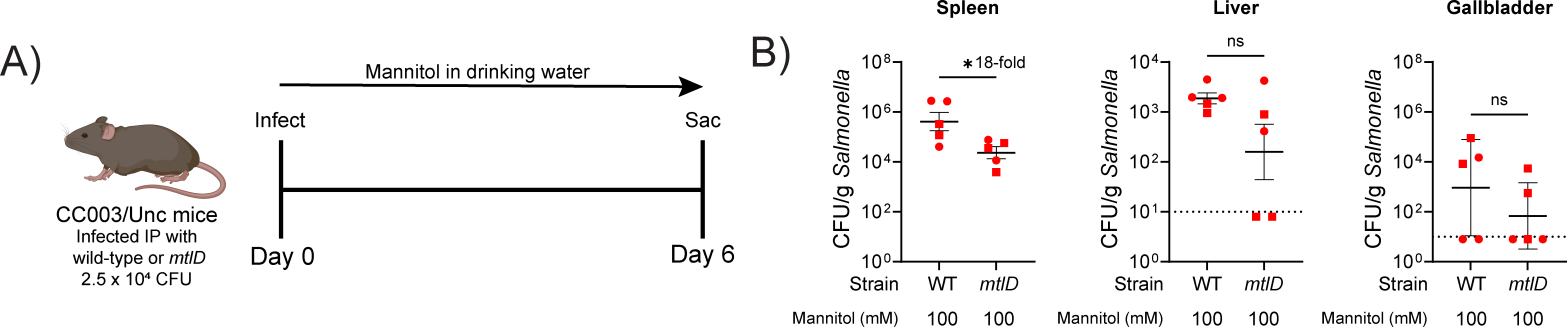
A *Salmonella enterica* serovar Typhi *mtlD* mutant is sensitive to mannitol in CC003/Unc mice. (**A, B**) Groups of five CC003/Unc mice were infected with 2.5 × 10^4^ CFU of either wild-type *Salmonella enterica* serovar Typhi (JSG4383, a *rpoS*-corrected version of Ty2) or Δ*mtlD6* mutant (AMS310) by the IP route. Mannitol was provided in drinking water immediately after infection at 100 mM for the duration of the experiment. On day 6, mice were sacrificed for enumeration of bacterial burden in the spleen, liver, and gallbladder. Male mice are indicated by squares, female mice are indicated by circles. Statistical significance was determined using two-tailed Student’s t test. *P < 0.05.

Based on our *in vitro* and *in vivo* findings above, we hypothesized that *mtlD* mutants of other pathogens could also be attenuated by mannitol during infection. To determine whether mannitol in drinking water could affect the human-adapted *Salmonella* serovar Typhi, we used a recently developed typhoidal mouse model (54). CC003/Unc mice were infected with serovar Typhi by the IP route to initiate a systemic infection and then provided mannitol in drinking water at 100 mM (Fig. 5A). This treatment led to an 18-fold reduction in burden of the *mtlD* mutant in the spleen but no change in the liver or gallbladder (Fig. 5B).

### Bacterial inoculum density and recovery from mannitol intoxication both affect IC_50_

**FIG 6:**
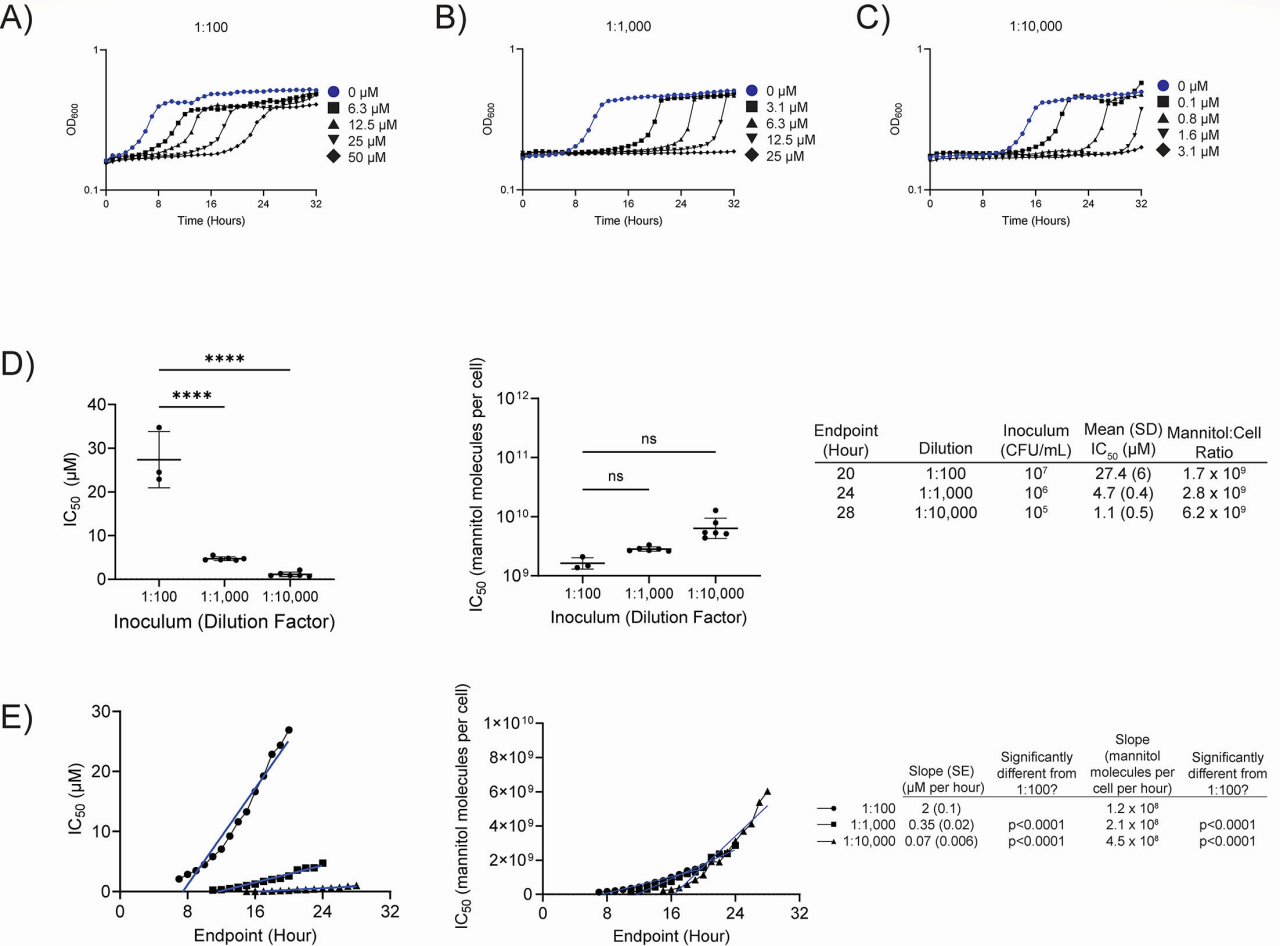
There is an inoculum effect and recovery from mannitol intoxication. **(A–C**) Growth of the *Salmonella enterica* serovar Typhimurium Δ*mtlD2* mutant (AMS302) in M9 + 5 mM fructose supplemented with mannitol at various concentrations. Concentrations are indicated to the right of each graph. The control (blue) contains no mannitol. The initial population of cells comes from an overnight culture washed and diluted 1:100 (1 × 10^7^ CFU/mL) (**A**), 1:1,000 (1 × 10^6^ CFU/mL) (**B**), or 1:10,000 (1 × 10^5^ CFU/mL) (**C**). Growth (OD_600_) was monitored for 32 h. (**D**) The IC_50_ of mannitol for the Δ*mtlD2* mutant (AMS302) grown in M9 fructose with units of µM (left) or number of mannitol molecules per cell (middle). The table (right) shows the time point that was chosen, the dilution, the inoculum (in CFU/mL), the mean IC_50_, and the mannitol-to-cell ratio for each dilution. Statistical significance in (D) was determined using two-tailed Student’s t test. (**E**) IC_50_ of mannitol for the Δ*mtlD2* mutant (AMS302) grown in M9 fructose using different time points for three inoculum dilutions (1:100, 1:1,000, 1:10,000), with units of µM (left) or number of mannitol molecules per cell (middle). Slopes were determined by linear regression analysis. Statistical significance was evaluated using Dunnett’s multiple comparisons test. ****P < 0.0001. The table (right) summarizes data and statistical analysis for the two graphs.

To determine the minimal inhibitory concentration (MIC) of mannitol, we grew each *mtlD* mutant in M9 fructose (or M9 Supp fructose) supplemented with mannitol at various concentrations. These assays revealed that intoxicated *mtlD* mutants eventually resume growth, a phenotype we refer to as recovery. The recovery phenotype occurs in a mannitol concentration-dependent manner: growth resumes faster when the mannitol concentration is lower (Fig. 6A). Additionally, beginning the growth assays with fewer cells appears to delay their entrance to the exponential phase of growth (Fig. 6B and C). Changes in MIC from changes in initial population size, referred to as inoculum effects, have been observed in the study of antibiotics (particularly beta-lactams) (63–67). Both phenotypes complicate the calculation of a mannitol MIC because the inhibitory concentration (IC_50_) changes as a function of both the initial population size and the time point selection.

We first determined the effect of inoculum size on MIC. The IC_50_ of a 1:100 diluted *ΔmtlD2* mutant (AMS302, ~ 10^7^ CFU/mL) is 27.4 µM after 20 h of growth (Fig. 6D). When the inoculum is diluted further, the IC_50_ is reduced 6-fold at a 1:1,000 dilution (~10^6^ CFU/mL) and 24-fold at a 1:10,000 dilution (~10^5^ CFU/mL), confirming an inoculum effect. It should be noted that the time point used for the calculation was 4 h later for each 10-fold dilution to compensate for the delay in reaching an equivalent OD_600_. The time point selected for IC_50_ readings does not abolish the inoculum effect, as we calculated the IC_50_ at hourly intervals for all cultures (Fig. 6E). No matter the time point chosen, the starting inoculum size affects the IC_50_. The IC_50_ changes over time are largely linear. This prompted us to calculate the IC_50_ as the number of mannitol molecules per cell. These plots were also linear over time, but the slopes for different inoculums are quite similar, ranging between 1.2 × 10^8^ molecules of mannitol per cell per hour for the highest concentration inoculum (1:100 dilution) to 4.5 × 10^8^ molecules of mannitol per cell per hour for the lowest concentration inoculum (1:10,000 dilution). We propose that this represents a rate for resolving the effects of, or processing, mannitol intoxication.

In Table 1, we present the inhibitory concentration (IC_50_) of *mtlD* mutants using either a 1:100 or 1:10,000 dilution from a washed overnight culture at the 20- or 28 h time point, respectively, in M9 fructose supplemented with mannitol. The time point used for M9 Supp was 10 or 14 h (for the 1:100 or 1:10,000 dilutions, respectively) to compensate for the faster growth rate and reduced lag phase of the strains. Using these criteria, the IC_50_ of mannitol for *mtlD* mutants of all species and strains tested is <50 µM. The inoculum effect and recovery phenotypes are observed in all *mtlD* mutants (Table 1; Fig. S3). Therefore, this may be a useful method of presenting inhibition data in situations where there are inoculum effects and recovery of growth.

**TABLE 1:** The IC_50_ of mannitol for *mtlD* mutants of *Escherichia coli*, *Salmonella enterica*, *Cronobacter sakazakii*, and *Pseudomonas aeruginosa* strains^*a*^

Media	Species	Strain background	Mutant	Strain	1:100 dilution		1:10,000 dilution	
					Mean IC_50_ (µM)	95% CI (µM)	Mean IC_50_ (µM)	95% CI (µM)
M9	*Salmonella enterica*	14028	*ΔmtlD1*	EFB004	28.3	16.0–51.7	ND	ND
M9	*Salmonella enterica*	14028	*ΔmtlD2*	AMS302	26.9	16.7–44.3	0.70	0.51–0.95
M9	*Salmonella enterica*	ST19	*ΔmtlD4*	AMS306	14.7	9.8–22.1	0.73	0.53–1.0
M9	*Salmonella enterica*	D23580	*ΔmtlD18*	AMS342	35.0	23.4–53.0	1.3	0.78–2.2
M9	*Salmonella enterica*	Paratyphi C	*ΔmtlD10*	AMS322	22.0	13.0–38.3	0.47	0.33–0.65
M9	*Escherichia coli*	K12	*ΔmtlD12*	AMS324	5.2	3.6–7.6	1.2	0.76–2.1
M9	*Escherichia coli*	700927	*ΔmtlD14*	AMS326	9.5	5.9–15.3	2.3	1.2–4.2
M9	*Cronobacter sakazakii*	MZ0686	*ΔmtlD22*	ECR005	17.7	13.2–23.6	3.6	2.6–4.9
M9	*Pseudomonas aeruginosa*	PAO1	*ΔmtlD22*	AMS353	14.1	7.5–27.2	5.3	3.0–9.4
M9	*Pseudomonas aeruginosa*	PAO1	*mtlD*::tet	unnamed	4.3	2.9–6.1	ND	ND
M9 Supp	*Salmonella enterica*	14028	*ΔmtlD2*	AMS302	27.2	18.5–40.5	1.6	1.1–2.2
M9 Supp	*Salmonella enterica*	Typhi	*ΔmtlD6*	AMS310	9.4	6.9–13.0	0.7	0.5–1.1
M9 Supp	*Salmonella enterica*	Paratyphi A	*ΔmtlD20*	AMS346	11.5	2.4–72.2	0.29	0.21–0.39
M9 Supp	*Escherichia coli*	UTI89	*ΔmtlD16*	AMS334	24.9	13.7–45.9	0.48	0.27–0.84
M9 Supp	*Salmonella enterica*	Paratyphi B	*ΔmtlD8*	AMS318	47.7	30.5–76.6	2.3	1.6–3.3

^*a*^ The IC_50_ calculations were performed on cultures grown in M9 minimal medium containing 5 mM fructose and a variable concentration of mannitol (M9) or in the same medium supplemented with 0.2% casamino acids and 1 mM tryptophan (M9 Supp) on three separate occasions (representative graphs are shown in Fig. S2). 95% confidence intervals are shown. The cultures were initiated with cells that had been previously grown overnight in LB, then washed, and diluted either 1:100 or 1:10,000. The IC_50_ was calculated for cultures grown from a 1:100 dilution in M9 using the 20 h time point; from a 1:10,000 dilution in M9 using the 28 h time point; from a 1:100 dilution in M9 Supp using the 10 h time point; and from a 1:10,000 dilution in M9 Supp using the 14 h time point. ND indicates not determined.

In Fig. S4, we present the data using a suppression index (68). Suppression index offers quantified values for evaluating the efficacy of mannitol-dependent inhibition of growth, in which the area under the OD-time curve of treated and untreated cells is compared. Because the *Salmonella mtlD* mutant recovers from mannitol over time (Fig. S4A), the suppression index is dependent on how long the growth measurements are performed. Therefore, we calculated the suppression index for 18 different time periods and plotted the results (Fig. S4B). As expected, the suppression index increases with increasing mannitol concentrations (Fig. S4C). The mannitol concentration that provides a suppression index of 0.5 is about 12.5 µM, comparable to the IC_50_ values obtained in Table 1.

## DISCUSSION

We became interested in sugar-phosphate toxicities (loosely defined) after characterizing a toxic metabolic intermediate within the fructose-asparagine utilization pathway (69). We then reviewed the literature surrounding other sugar-phosphate toxicities (34) and tested the induction of seven of these toxicities for attenuation of *Salmonella* in the murine gastrointestinal tract (53). Of the seven, the provision of rhamnose to a *rhaD* mutant, arabinose to an *araD* mutant, or mannitol to a *mtlD* mutant, caused severe attenuation of *Salmonella* in the gastrointestinal tract (53). We hypothesize that any of these three enzymes could be used as a therapeutic target for the treatment of *Salmonella*-mediated gastroenteritis. However, of these three we suspect that MtlD is the most promising therapeutic target, primarily because as we show here, mannitol can reach the *mtlD* mutant of *Salmonella* at systemic sites. In contrast, injection of rhamnose does not inhibit a *rhaD* mutant at systemic sites (Fig. S5). We have not yet tested the effect of arabinose on an *araD* mutant at systemic sites. Because *Salmonella* is an intracellular pathogen, we were surprised that mannitol administered orally or IP to mice could reach *Salmonella* in the spleen and liver. However, there are two previous publications demonstrating that, at least in tissue culture cells, mannitol in the growth medium is metabolized by intracellular *Salmonella* (70, 71). IV injection of mannitol has also been shown to attenuate a *mtlD* mutant of *S. aureus* in the kidneys and liver (41). How mannitol gains entry to eukaryotic cells is not known.

Another advantage of MtlD as a therapeutic target is that the safety profile and pharmacokinetics of mannitol are well known. Mannitol is a natural product, synthesized by plants and fungi as a compatible solute to regulate osmolarity, and also as a storage molecule and a redox protectant (42). In humans, mannitol is metabolically inert with 80% of the mannitol injected intravenously being secreted into the urine within 3 h (72). The osmotic properties of mannitol enable its use in medicine as an osmotic diuretic to reduce intracranial pressure/cerebral edema, to reduce intraocular pressure, or to promote diuresis in the oliguric phase of acute renal failure (OSMITROL, NDC0338-0357-03) (73). Mannitol has also found application in the respiratory tract, both as a diagnostic for asthma and as a therapeutic to enhance mucociliary clearance in cystic fibrosis patients (reviewed in (74)). The host microbiota appears to metabolize a significant percentage of orally consumed mannitol, reducing the efficiency of uptake via the oral route (72). While mannitol is clearly safe for humans, there are some caveats. Some polyols can cause osmotic diarrhea, intestinal bloating, or flatulence when consumed in high quantities (especially glucitol, also known as sorbitol) (75–77). The US Food and Drug Administration requires that any human food that may result in more than 20 g of mannitol ingestion per day be labeled as potentially having a laxative effect. Thus, identifying the lowest concentration of mannitol, and most effective route of delivery, will be important if this strategy is to be used to treat infections.

Overall, our data indicate that a MtlD inhibitor coupled with mannitol may be an effective therapeutic strategy in combating gastroenteritis or systemic infection caused by the nontyphoidal *Salmonella* serovars including the invasive nontyphoidal serovars that have recently emerged in Africa. It is likely that the typhoidal serovars could also be treated with this strategy. MtlD mutants of serovars Typhi, Paratyphi A, B, and C are all similar to serovar Typhimurium with regard to mannitol sensitivity. Additionally, we used a new mouse model that is permissive to serovar Typhi infection to demonstrate that mannitol can reduce the quantity of a serovar Typhi *mtlD* mutant in the spleen. Unfortunately, these mice are expensive and slow to reproduce so we have only tried one dose of mannitol by one route. The route and concentration chosen, 100 mM in drinking water, is likely not optimal. When more mice become available, we would like to test the hypothesis that a higher concentration of mannitol in the drinking water, or via the IV route, could more thoroughly eliminate serovar Typhi from the mice.

We constructed a *mtlD* mutant of *Cronobacter sakazakii* and confirmed that it is sensitive to mannitol. This organism can contaminate powdered infant formula and then cause lethal infections in the neonates fed the formula. The administration of mannitol and a MtlD inhibitor may be able to treat these infections, as well.

MtlD is highly conserved among the *Escherichia*, *Salmonella*, *Cronobacter*, *Streptococcus*, *Vibrio*, the CRE pathogens (carbapenem-resistant Enterobacteriaceae), and most of the ESKAPE pathogens including *Enterococcus*, *Staphylococcus, Klebsiella*, *Pseudomonas*, and *Enterobacter* (53). Gene presence is likely to predict drug effectiveness as MtlD is highly conserved among the genera listed above (>50% identity) and X-ray crystal structures of MtlD from a Gram-positive organism, *S. aureus*, and a Gram-negative organism, *Shigella flexneri*, reveal highly conserved NAD^+^ and mannitol-binding residues as well as the catalytic triad (Lys, Asn, Asn) (41, 78). MtlD is found in only 2% of the Bacteroidota and 40% of the Firmicutes, thus inhibitors of MtlD would likely spare much of the normal microbiota in the gastrointestinal tract (53). MtlD is a narrow-spectrum target, but not too narrow to limit utility.

Our efforts to quantify the MIC of mannitol for *mtlD* mutants were complicated by both an inoculum effect and a recovery phenotype. Inoculum effects are changes in inhibitory concentrations of compounds (e.g., antibiotics) due to changes in the initial bacterial population (63–67, 79). The IC_50_ of mannitol is reduced by diluting the initial population. These inoculum effects are greatly reduced by presenting the IC_50_ as molecules of mannitol per cell rather than simply mannitol concentration (Fig. 6D). The second complication is that the bacteria recover from intoxication over time. Thus, choosing a time point for the IC_50_ calculation has large effects on the result. When we calculated the IC_50_ at every time point (hourly), we noted a linear relationship between IC_50_ and time. The slope of the line in molecules of mannitol per cell per hour provides what we propose to be a processing rate for the effects of mannitol toxicity (Fig. 6E). “Processing” could represent the elimination of Mtl-1P either by cleavage, conversion to another molecule, or efflux from the cell (80). Repairing damage caused by Mtl-1P accumulation may also be necessary. To our knowledge, antibiotic-challenged cells do not exhibit recovery phenotypes. The unique nature of this phenotype may prompt the need for alternative approaches in quantifying toxicity (i.e., processing rate rather than MICs or suppression indices). The underlying mechanisms of both intoxication and recovery are under active investigation in our lab and could inform the application of a future therapeutic.

## MATERIALS AND METHODS

### Bacterial strains and media

Strains and plasmids used in this study are listed in Table S1. Bacteria were routinely grown in LB or on LB agar (1.5% w/v). Minimal media (M9) contained 1× M9 salts, 2 mM MgSO_4_, 0.1 mM CaCl_2_, 0.01 mM thiamine, and trace elements (81). M9 Supp is M9 with casamino acids (final concentration 0.2%) and tryptophan (final concentration 1 mM). Sugars were supplemented into the media at the designated concentration in the text. Antibiotics were used at the following final concentrations: kanamycin (kan) at 50 µg/mL, chloramphenicol (cam) at 30 µg/mL, ampicillin (amp) at 100 µg/mL, and gentamicin at 10 or 50 µg/mL. Diaminopimelic acid was used at a final concentration of 100 µM. Sucrose was used at a final concentration of 10%. Anhydrotetracycline (AHT) was used at a final concentration of 0.5 µg/mL.

### Construction of mutants

Primers used in this study are listed in Table S2. Deletions of *mtlA* and *mtlD* in *Salmonella enterica, Escherichia coli, Cronobacter sakazakii,* and *Pseudomonas aeruginosa* were constructed using allelic exchange. Each mutation was made by a strain-specific suicide vector made with Gibson assembly in the vector backbone pFOK (*Salmonella*, *Escherichia, Cronobacter*) or pEX18 (*Pseudomonas*). Each plasmid had two inserted fragments (the upstream and downstream regions of the target gene, to create an in-frame deletion) with overhangs homologous to the first or last 30 nucleotides of the deleted gene. The final product encodes the first and last 10 amino acids in each gene. We identified regions upstream, downstream, and within the *mtl* locus to act as sites of conserved overlap homology (sequences were conserved in all strains within the species at those specific sites). This reduced the number of primers needed for construction of mutations in different strains. However, for each strain, a unique suicide plasmid was made. The *Salmonella* upstream *mtlA* fragment was amplified with primers BA4111 and BA4113 and downstream fragment with primers BA4114 and BA4112. The upstream *mtlD* fragment was amplified with primers BA4111 and BA4115 and downstream fragment with primers BA4116 and BA4112. The *E. coli* upstream *mtlA* fragment was amplified with primers BA4127 and BA4128 and downstream fragment with primers BA4129 and BA4120. The upstream *mtlD* fragment was amplified with primers BA4117 and BA4118 and downstream fragment with primers BA4119 and BA4120. The *Cronobacter* upstream *mtlA* fragment was amplified with primers BA4143 and BA4144 and downstream fragment with primers BA4145 and BA4148. The upstream *mtlD* fragment was amplified with primers BA4143 and BA4146 and downstream fragment with primers BA4147 and BA4148. The *P. aeruginosa* upstream *mtlD* fragment was amplified with primers BA4138 and BA4136 and downstream fragment with primers BA4137 and BA4135.

The pFOK vector was linearized by PCR with primers BA3875 and BA3876. The pEX18 vector was linearized with primers BA4130 and BA4131. Vector and fragments were purified by gel extraction, quantified by nanodrop, and assembled according to the manufacturer’s instructions (NEB cat # E5510). Product was transformed into TransforMax EC100D pir+ *E. coli* by electroporation (Lucigen ECP09500), selecting LB kan at 50 µg/mL (pFOK) or LB gent at 10 µg/mL (pEX18). Plasmids were confirmed by PCR, purified from EC100D pir+ cells, and moved into mating strain Jke201 by electroporation (LB DAP kan or LB DAP gent at 10 µg/mL). Allelic exchange was performed by mating Jke201+ plasmid with a recipient strain on LB DAP. Exconjugants were isolated on LB kan or LB gent at 50 µg/mL. Isolates were outgrown without selection in LB and dilution plated on LB AHT sucrose (pFOK) or LB sucrose (pEX18). Individual colonies were screened for loss of vector resistance to identify isolates in which the vector has recombined out of the chromosome. Mutations were confirmed by PCR.

### MtlD complementation plasmid

The complementation plasmid of MtlD was constructed in the low copy number vector pWSK29 (amp^r^) (82). The *mtlD* gene of *Salmonella* strain 14028 was amplified by PCR with primers BA4123 and BA4124. The PCR product was cloned into pCR2.1 (TOPO, kan^r^). This plasmid was digested with EcoRI to remove the *mtlD* insert and ligated into the EcoRI site of pWSK29 with T4 ligase, transformed into competent *E. coli*, selecting on LB amp. Isolates were screened for insertion and orientation using BA2473 and BA4124. The confirmed plasmid (pAMS394) and vector (pWSK29) were transformed into 14028 *ΔmtlD2* (AMS302) by electroporation. Isolates were selected and maintained on LB amp.

### Growth assays

Growth was measured over time in the Spectramax i3x (Molecular Devices) in flat, clear-bottom plates (Corning, catalog # 3370), without shaking, at 37°C. Readings of the optical density at 600 nm (OD_600_), using a path length of 0.33 cm, were taken at the times indicated in each figure. Overnight cultures of strains were washed and resuspended in water, then inoculated into designated media at a dilution of 1:100 (2 µL of culture and 198 µL of media). For further dilutions, washed cultures were serially diluted in water 10-fold before inoculating. A breathe-easy membrane film (Sigma, catalog # Z380059) was placed over the top of each plate to allow for gas exchange. All growth assays were performed on at least three separate occasions.

### Minimum inhibitory concentration

Inhibitory concentration (IC_50_) was determined for each strain using growth assays in M9 or M9 Supp (M9 + 0.2% casamino acids + 1 mM tryptophan) + 5 mM fructose. Mannitol was added in a series of concentrations varying twofold. Each strain was grown overnight in LB, washed and diluted in water, then inoculated into the media. Growth was measured every hour (measuring OD_600_) at each time designated in each experiment. IC_50_ was calculated by nonlinear regression analysis, using normalized growth, bracketed by a no mannitol control representing maximum growth (100%) and the no growth control (0%) at the specified time. In each assay, the highest concentration used was sufficient to prevent growth of the culture (recovery) for the time point used in the calculation. All MIC assays were done on at least three independent occasions.

### Sex of mice

In a previous publication, no differences in *mtlD* phenotype of *Salmonella enterica* serovar Typhimurium were noted based on the sex of the mice (53). Therefore, in this report only female mice are used with serovar Typhimurium. For the experiment with serovar Typhi both sexes of CC003/Unc mice were used.

### Systemic infections with *S. enterica* serovar Typhimurium

Six-to-eight-week-old C57BL/6 and Swiss Webster mice were acquired from Jackson Labs and Taconic Farms, respectively. Overnight cultures of strains designated for each experiment were washed and resuspended in water. After diluting to the desired concentration, mice were infected by IP injection in a total volume of 200 µL. Mice were monitored daily for weight loss and early removal criteria, including weight loss >20%.

In the competition experiment using C57BL/6J mice, where specified, mice were treated, or not, with mannitol by either IP injection or drinking water (Fig. 2). For IP treatment, one dose per day for three (days 1–3) of 1 g/kg were delivered to each mouse. For drinking water treatment, mannitol was supplemented into their drinking water to specified final concentrations, beginning after infection (day 0). Mice were euthanized on day 4. Homogenized liver and spleen were dilution plated on LB kan and LB cam for determining wild-type and mutant burdens. Competitive index (CI) was calculated as the ratio of mutant to wild-type divided by the initial mutant-to-wild-type ratio.

In the survival study using C57BL/6J mice, where specified, mice were treated or not with mannitol in drinking water (Fig. 2). For drinking water treatment, mannitol was supplemented into their drinking water to the specified final concentrations beginning after infection (day 0). On day 4, half of each group was euthanized for determining bacterial burden. Homogenized liver and spleen were plated on LB. The remaining mice were monitored and euthanized upon reaching removal criteria.

In the survival studies using Swiss Webster mice, where specified, mannitol was provided in drinking water (Fig. 3). For drinking water treatment, mannitol was supplemented into their drinking water to the specified final concentrations beginning after infection (day 0). After infection, mice were monitored using the same criteria. Mice reaching the end of the study (day 21) were euthanized for determining *Salmonella* burden in the liver and spleen. Homogenized organs were plated on LB.

In the competition experiment using C57BL/6J mice testing rhamnose-dependent fitness, the experiment was performed identically as the mannitol experiment above except different strains were used and rhamnose was used instead of mannitol (Fig. S5). Rhamnose was delivered IP at 1 g/kg per day for 3 days and drinking water treatment containing rhamnose at 100 mM was provided beginning after infection.

### Gastroenteritis infections with *S. enterica* serovar Typhimurium

Six-to-eight-week-old Swiss Webster mice were acquired from Taconic Farms (Fig. 4). Mice were pretreated with 20 mg in 200 µL of streptomycin, delivered by oral gavage (day −1). One day later, mice were infected with *Salmonella* by oral gavage in 200 µL water (day 0). Where specified, mice not treated or treated with mannitol by drinking water, supplemented into their drinking water to specified final concentrations beginning after infection (day 0). Feces were collected daily for 4 days, homogenized, and plated on XLD for quantification. On day 5, mice were euthanized, and ceca were collected for determining *Salmonella* burden. Proximal colon was collected, stored in formalin, and sent to HistoWiz (Brooklyn, NY, USA) for histopathology, which was analyzed without knowledge of group conditions (e.g., control vs treatment).

### Systemic infections with *S. enterica* serovar Typhi

Both sexes of CC003/Unc mice were bred in-house and used at 7 weeks of age. 2.5 × 10^4^ CFU of *Salmonella enterica serovar* Typhi were delivered IP in a total volume of 200 µL of PBS (Fig. 5). Mice were provided 100 µM mannitol in their drinking water beginning immediately after infection (day 0). On day 6, mice were euthanized, and organs (liver, spleen, and gallbladder) were harvested for enumeration of CFU by plating on LB agar.

### Animal assurance

All animal work was performed using protocols approved by our Institutional Animal Care and Use Committee (IACUC; OSU 2009A0035) and in accordance with the relevant guidelines set forth in the Guide for the Care and Use of Laboratory Animals (83).

### Quantification of mannitol in LB and mouse chow

d-Mannitol was purchased from Sigma-Aldrich (MO, USA). d-Mannitol(^13^C_6_) was purchased from Cambridge Isotope Laboratories (MA, USA). Optima LC/MS grade formic acid, water, and acetonitrile were purchased from Fisher Scientific (MA, USA). Precellys lysing kit was purchased from Bertin Technologies (France).

For quantification of mannitol in mouse chow, 20 mg of sample and 1 mL DCM/methanol/water (3:2:1; v/v/v) extract solution were added to 2 mL Precellys lysing kit (Bertin, France). Each sample was homogenized at 6800 RPM for four cycles (30 s per cycle with 45 s pause) using Bertin Precellys Homogenizer (Bertin, France). The homogenized sample was sonicated in a water bath for 10 min at room temperature and followed by centrifugation at 10,000 rcf for 5 min. To minimize matrix effect, 2 µL of aqueous phase extract was diluted 50 times with water and spiked with 1 ppm internal standard. External calibration was prepared in water with spiked internal standard at 1 ppm. Five microliters of calibration and samples were analyzed by LC-MS/MS.

For quantification of mannitol in LB, 20 µL broth and 1 mL DCM/methanol/water (3:2:1; v/v/v) extract solution was added to a 2 mL Eppendorf tube. The sample was vortexed for 20 s, sonicated in water bath for 10 min, and followed by centrifugation at 10,000 rcf for 5 min at room temperature. The aqueous phase was transferred out and diluted 20 times before preparing the standard addition. The individual calibration was prepared by spiking 0.5, 1, 2, 3, and 4 ppm of d-mannitol in the diluted aqueous extract. The internal standard of d-mannitol(^13^C_6_) was spiked in each calibration levels at 1 ppm. Finally, 5 μL of the standard addition levels were analyzed by LC-MS/MS. Both calibration curves and samples were analyzed in triplicates.

The quantification was carried out on a Vanquish UHPLC coupled to an Orbitrap Exploris 480 mass spectrometer (Thermo Fisher, MA, USA). The analytes were separated on a Accucore C18 2.6 µm 2.1 × 100 mm column using the binary solvents of water with 0.1% formic acid (v/v) (solvent A) and acetonitrile with 0.1% formic acid (v/v) (solvent B). The gradient was 0–1 min, 2% B; 1–3 min, 2–5% B; 3–5 min, 5–50% B; 5–6 min, 50–95% B; 6–8 min, holding at 95% B; 8–8.01 min, 95–2% B; 8.01–10 min, holding at 2% B. The flow rate of 0.3 mL/min. The following mass spectrometer instrument settings were used: ion source = H-ESI; positive ion = 3,500 V; sheath gas = 35; aux gas = 7; ion transfer tube temperature = 320°C; vaporizer temperature = 275°C; HCD collision energy = 60%; RF lens = 60%. The mannitol (205.0683 m/z) and mannitol(^13^C_6_) (211.0884 m/z) were detected by tMS^2^ mode between 0 and 9 min. Both external calibration and standard addition curves demonstrated great linearity with R^2^ > 0.99.

## SUPPLEMENTAL MATERIAL

Figure S1 jb.00480-24-s0001.tif CFU correlates to OD600.

Figure S2 jb.00480-24-s0002.tif Growth curves of numerous strains and mutants under different conditions.

Figure S3 jb.00480-24-s0003.tif Recovery from mannitol intoxication is conserved among mtlD mutants of different bacterial species and strains.

Figure S4 jb.00480-24-s0004.tif Suppression index for mannitol intoxicated Salmonella mtlD mutant.

Figure S5 jb.00480-24-s0005.tif Treatment of mice infected with Salmonella rhaD mutant using IP rhamnose.

Table S1 jb.00480-24-s0006.docx Strains and plasmids used.

Table S2 jb.00480-24-s0007.docx Primers used.

ASM does not own the copyrights to Supplemental Material that may be linked to, or accessed through, an article. The authors have granted ASM a non-exclusive, world-wide license to publish the Supplemental Material files. Please contact the corresponding author directly for reuse.
