# Supplementary figures and images for: MtlD as a therapeutic target for intestinal and systemic bacterial infections
Source: J Bacteriol. 2024 Dec 27;207(1):e00480-24. doi: 10.1128/jb.00480-24 (PMC11784389; doi:10.1128/jb.00480-24)

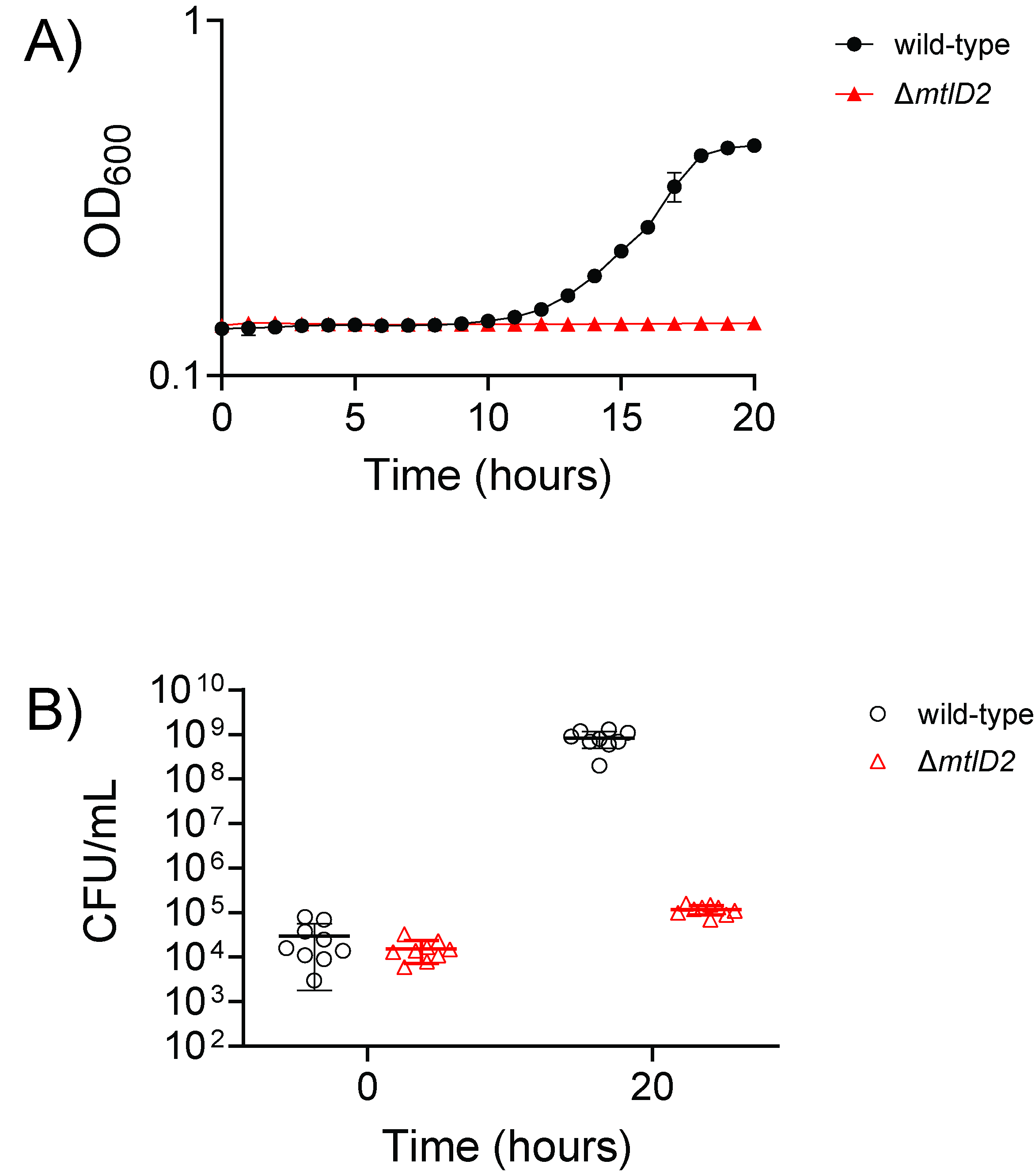

Supplement: Figure S1 — CFU correlates to OD600. [file jb.00480-24-s0001.tif]

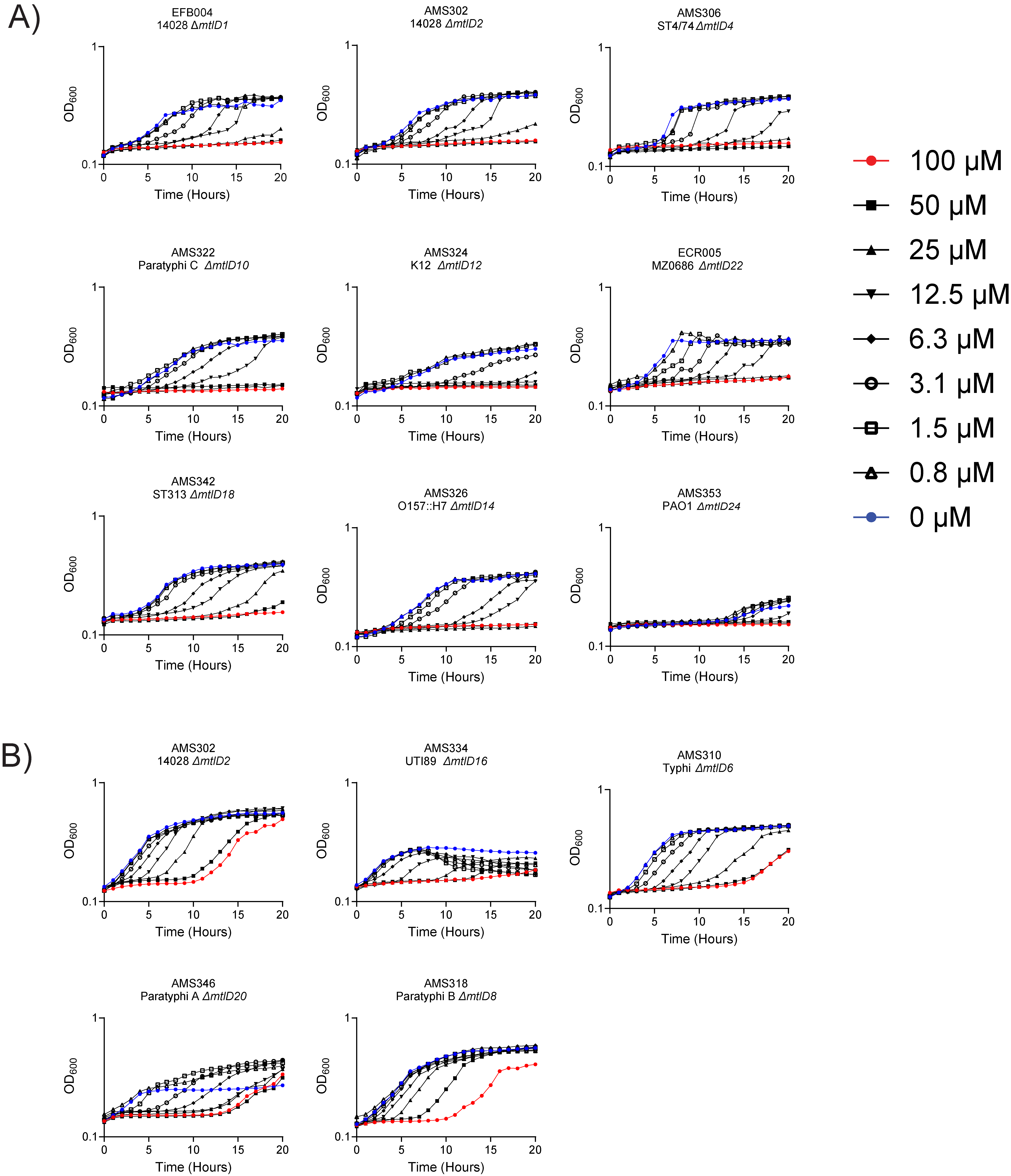

Supplement: Figure S3 — Recovery from mannitol intoxication is conserved among mtlD mutants of different bacterial species and strains. [file jb.00480-24-s0003.tif]

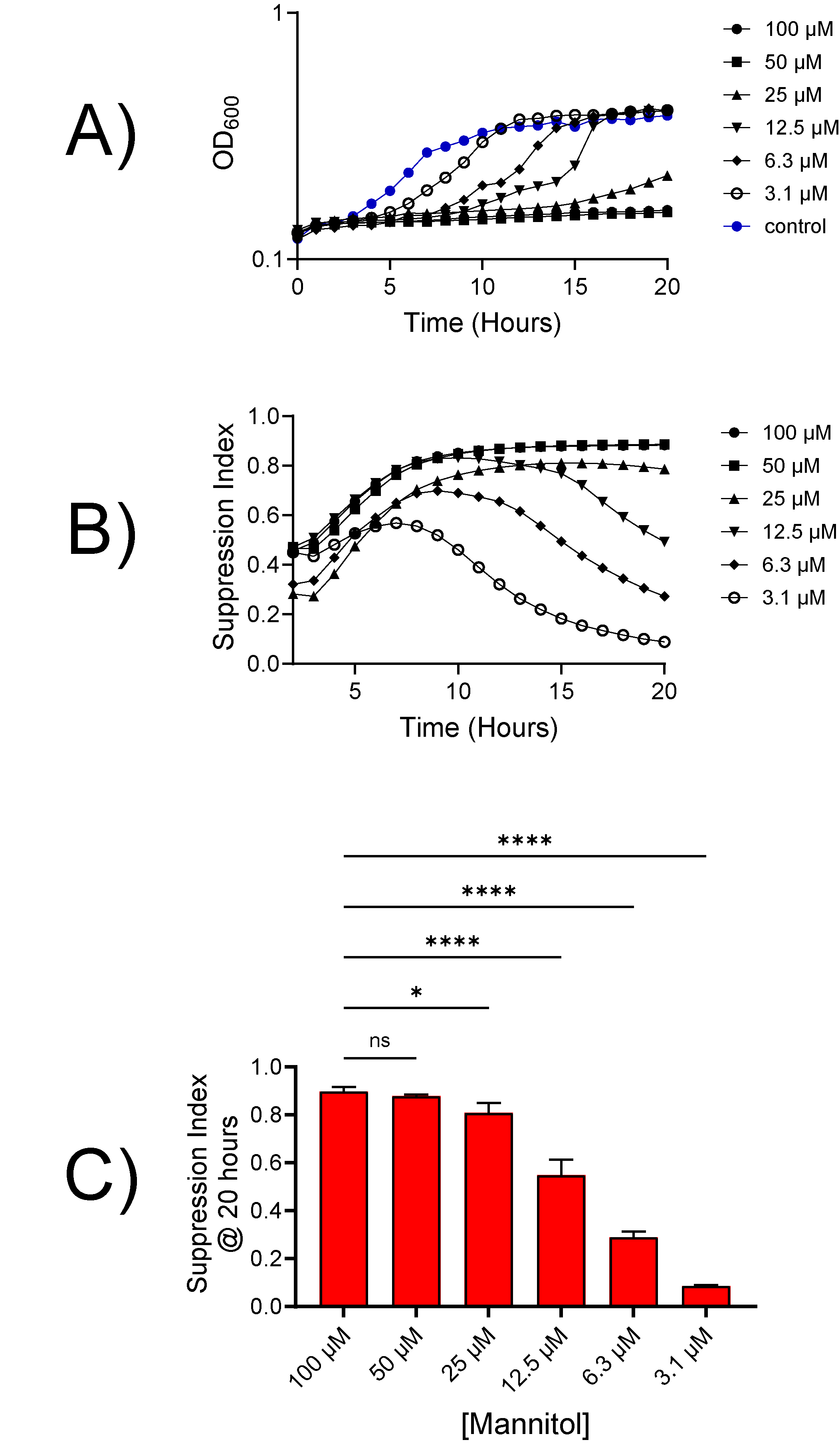

Supplement: Figure S4 — Suppression index for mannitol intoxicated Salmonella mtlD mutant. [file jb.00480-24-s0004.tif]

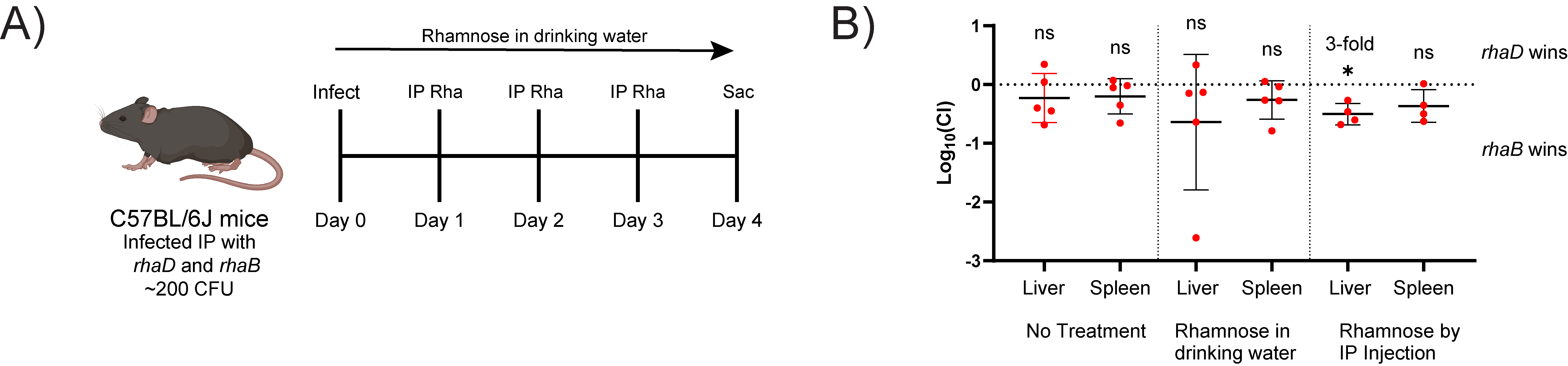

Supplement: Figure S5 — Treatment of mice infected with Salmonella rhaD mutant using IP rhamnose. [file jb.00480-24-s0005.tif]
