# Supplementary material for: MtlD as a therapeutic target for intestinal and systemic bacterial infections
Source: J Bacteriol. 2024 Dec 27;207(1):e00480-24. doi: 10.1128/jb.00480-24 (PMC11784389; doi:10.1128/jb.00480-24)
Supplement: Table S1 — Strains and plasmids used. [file jb.00480-24-s0006.docx]

Table S1. Strains and plasmids used in this study.

| **Strain** | **Genotype or Description** | **Source, Construction, or Reference** |
| --- | --- | --- |
| ATCC 14028 (14028) | *Salmonella* *enterica*  serovar Typhimurium | American Type Culture Collection (ATCC) |
| ST4/74 | *Salmonella* *enterica* serovar Typhimurium | ^1^ |
| JSG4383 | *Salmonella* *enterica* serovar Typhi Ty2 (*rpoS*^+^) | ^2,3^ |
| D23580 | *Salmonella enterica* serovar Typhimurium ST313 lineage 2 | Jay Hinton ^4^ |
| MZ1299 (SGSC4902) | *Salmonella enterica* serovar Paratyphi A | Michael McClelland |
| MZ0955 (SGSC4150) | *Salmonella enterica* serovar Paratyphi B | Michael McClelland ^5^ |
| MZ0875 (SGSC2290) | *Salmonella enterica* serovar Paratyphi C | Michael McClelland |
| MG1655 | *Escherichia coli* K-12 | *E. coli* Genetic Stock Center |
| 700927 | *Enterohemorrhagic Escherichia coli* O157:H7 (EHEC) | ATCC |
| UTI89 | Uropathogenic *Escherichia coli* (UPEC) | Sheryl Justice |
| Jke201 | Mating strain of *E. coli*, see reference ^6^ for full genotype and description | Gift from Dirk Bumann, ^6^ |
| PAO1 | *Pseudomonas aeruginosa* strain PAO1 | Daniel Wozniak |
| MZ0686 | *Cronobacter sakazakii* | Michael McClelland |
| JLD1214 | 14028 *IG(pagC-STM14_1502)*::cam | ^7^ |
| EFB004 | 14028 *ΔmtlD1* | ^8^ |
| EFB036 | 14028 *mtlA1::cam* | ^8^ |
| EFB063 | *14028 rhaB1*::kan | ^8^ |
| EFC015 | 14028 *rhaD1*::cam | ^8^ |
| AMS276 | 14028 *ΔmtlD1 IG(pagC-STM14_1502)*::cam. P22 transduction of *IG(pagC-STM14_1502)*::cam from JLD1214 to EFB004 | This Study |
| AMS300 | 14028 *ΔmtlA2* | This Study |
| AMS302 | 14028 *ΔmtlD2* | This Study |
| AMS304 | ST4/74 *ΔmtlA4* | This Study |
| AMS306 | ST4/74 *ΔmtlD4* | This Study |
| AMS308 | JSG4383 *ΔmtlA6* | This Study |
| AMS310 | JSG4383 *ΔmtlD6* | This Study |
| AMS316 | MZ0955 *ΔmtlA8* | This Study |
| AMS318 | MZ0955 Δ*mtlD8* | This Study |
| AMS320 | MZ0875 *ΔmtlA10* | This Study |
| AMS322 | MZ0875 *ΔmtlD10* | This Study |
| AMS324 | MG1655 *ΔmtlD12* | This Study |
| AMS326 | 700927 *ΔmtlD14* | This Study |
| AMS328 | MG1655 *ΔmtlA12* | This Study |
| AMS330 | 700927 *ΔmtlA14* | This Study |
| AMS332 | UTI89 *ΔmtlA16* | This Study |
| AMS334 | UTI89 *ΔmtlD16* | This Study |
| AMS340 | D23580 *ΔmtlA18* | This Study |
| AMS342 | D23580 *ΔmtlD18* | This Study |
| AMS344 | MZ1299 *ΔmtlA20* | This Study |
| AMS346 | MZ1299 *ΔmtlD20* | This Study |
| AMS353 | PAO1 *ΔmtlD24* | This Study |
| *mtlD*::tet | PAO1 *mtlD*::tet | ^9^ |
| ECR003 | MZ0686 Δ*mtlA22* | This Study |
| ECR005 | MZ0686 Δ*mtlD22* | This Study |
| **Plasmid** | **Genotype or Description** | **Source, Construction, or Reference** |
| pFOK | Suicide vector backbone for allelic exchange. kan^r^ | Dirk Bumann ^10^ |
| pWSK29 | pSC101 cloning vector. amp^r^ | ^11^ |
| pAMS394 | pWSK29-MtlD_14028_ | This Study |
| pAMS370 | Suicide vector for construction of 14028 *mtlA* | This Study |
| pAMS371 | Suicide vector for construction of 14028 *mtlD* | This Study |
| pAMS373 | Suicide vector for construction of ST4/74 *mtlA* | This Study |
| pAMS374 | Suicide vector for construction of ST4/74 *mtlD* | This Study |
| pAMS375 | Suicide vector for construction of JSG4383 *mtlA* | This Study |
| pAMS376 | Suicide vector for construction of JSG4383 *mtlD* | This Study |
| pAMS377 | Suicide vector for construction of MZ1299 *mtlA* | This Study |
| pAMS378 | Suicide vector for construction of MZ1299 *mtlD* | This Study |
| pAMS379 | Suicide vector for construction of MZ0955 *mtlA* | This Study |
| pAMS380 | Suicide vector for construction of MZ0955 *mtlD* | This Study |
| pAMS381 | Suicide vector for construction of MZ0875 *mtlA* | This Study |
| pAMS382 | Suicide vector for construction of MZ0875 *mtlD* | This Study |
| pAMS383 | Suicide vector for construction of MG1655 *mtlD* | This Study |
| pAMS384 | Suicide vector for construction of 700927 *mtlD* | This Study |
| pAMS385 | Suicide vector for construction of MG1655 *mtlA* | This Study |
| pAMS386 | Suicide vector for construction of 700927 *mtlA* | This Study |
| pAMS387 | Suicide vector for construction of UTI89 *mtlA* | This Study |
| pAMS388 | Suicide vector for construction of UTI89 *mtlD* | This Study |
| pAMS403 | Suicide vector for construction of PAO1 *mtlD* | This Study |
| pECR001 | Suicide vector for construction of MZ0686 *mtlA* | This Study |
| pECR003 | Suicide vector for construction of MZ0686 *mtlD* | This Study |

**References**

1. Richardson, E.J., B. Limaye, H. Inamdar, A. Datta, K.S. Manjari, G.D. Pullinger, N.R. Thomson, R.R. Joshi, M. Watson, and M.P. Stevens, *Genome Sequences of Salmonella enterica Serovar Typhimurium, Choleraesuis, Dublin, and Gallinarum Strains of Well- Defined Virulence in Food-Producing Animals.* Journal of Bacteriology, 2011. **193**(12): p. 3162-3163.

2. Burda, W.N., K.E. Brenneman, A. Gonzales, and R. Curtiss, *Conversion of RpoS Attenuated Salmonella enterica Serovar Typhi Vaccine Strains to RpoS+ Improves Their Resistance to Host Defense Barriers.* mSphere, 2018. **3**(1): p. 10.1128/msphere.00006-18.

3. Sandala, J.L., B.W. Eichar, L.G. Kuo, M.M. Hahn, A.K. Basak, W.M. Huggins, K. Woolard, C. Melander, and J.S. Gunn, *A dual-therapy approach for the treatment of biofilm-mediated Salmonella gallbladder carriage.* PLOS Pathogens, 2021. **16**(12): p. e1009192.

4. Kingsley, R.A., C.L. Msefula, N.R. Thomson, S. Kariuki, K.E. Holt, M.A. Gordon, D. Harris, L. Clarke, S. Whitehead, V. Sangal, K. Marsh, M. Achtman, M.E. Molyneux, M. Cormican, J. Parkhill, C.A. MacLennan, R.S. Heyderman, and G. Dougan, *Epidemic multiple drug resistant Salmonella Typhimurium causing invasive disease in sub-Saharan Africa have a distinct genotype.* Genome Res, 2009. **19**(12): p. 2279-87.

5. Swearingen, M.C., S. Porwollik, P.T. Desai, M. McClelland, and B.M.M. Ahmer, *Virulence of 32 Salmonella Strains in Mice.* PLOS ONE, 2012. **7**(4): p. e36043.

6. Harms, A., M. Liesch, J. Körner, M. Québatte, P. Engel, and C. Dehio, *A bacterial toxin-antitoxin module is the origin of inter-bacterial and inter-kingdom effectors of Bartonella.* PLOS Genetics, 2017. **13**(10): p. e1007077.

7. Ali, M.M., D.L. Newsom, J.F. González, A. Sabag-Daigle, C. Stahl, B. Steidley, J. Dubena, J.L. Dyszel, J.N. Smith, Y. Dieye, R. Arsenescu, P.N. Boyaka, S. Krakowka, T. Romeo, E.J. Behrman, P. White, and B.M.M. Ahmer, *Fructose-Asparagine Is a Primary Nutrient during Growth of Salmonella in the Inflamed Intestine.* PLOS Pathogens, 2014. **10**(6): p. e1004209.

8. Boulanger, E.F., A. Sabag-Daigle, M. Baniasad, K. Kokkinias, A. Schwieters, K.C. Wrighton, V.H. Wysocki, and B.M.M. Ahmer, *Sugar-Phosphate Toxicities Attenuate Salmonella Fitness in the Gut.* Journal of Bacteriology, 2022. **0**(0): p. e00344-22.

9. Jacobs, M.A., A. Alwood, I. Thaipisuttikul, D. Spencer, E. Haugen, S. Ernst, O. Will, R. Kaul, C. Raymond, R. Levy, L. Chun-Rong, D. Guenthner, D. Bovee, M.V. Olson, and C. Manoil, *Comprehensive transposon mutant library of Pseudomonas aeruginosa.* Proceedings of the National Academy of Sciences, 2003. **100**(24): p. 14339-14344.

10. Cianfanelli, F.R., O. Cunrath, and D. Bumann, *Efficient dual-negative selection for bacterial genome editing.* BMC Microbiology, 2020. **20**(1): p. 129.

11. Wang, R.F. and S.R. Kushner, *Construction of versatile low-copy-number vectors for cloning, sequencing and gene expression in Escherichia coli.* Gene, 1991. **100**: p. 195-9.
