# Supplementary material for: MtlD as a therapeutic target for intestinal and systemic bacterial infections
Source: J Bacteriol. 2024 Dec 27;207(1):e00480-24. doi: 10.1128/jb.00480-24 (PMC11784389; doi:10.1128/jb.00480-24)
Supplement: Table S2 — Primers used. [file jb.00480-24-s0007.docx]

Table S2. Primers used in this study.

| **Primer** | **Sequence** | **Description** |
| --- | --- | --- |
| BA2473 | CGGCATTCGCCATTCAGGCTGCCTC | Verification of pWSK29 insertions |
| BA3875 | ATCGAATTCCTGCAGCCCGGGGGATCCACT | pFOK linearization |
| BA3876 | ATCAAGCTTATCGATACCGTCGACCTCGAG | pFOK linearization |
| BA4111 | CTCGAGGTCGACGGTATCGATAAGCTTGATGGGATATCGACATAAGGGGGATTGTAACGT | For constructing *Salmonella* specific suicide plasmids |
| BA4112 | AGTGGATCCCCCGGGCTGCAGGAATTCGATGATCGCTCAGGCGTTTAATTTCGTTTTTTT | For constructing *Salmonella* specific suicide plasmids |
| BA4113 | TTAAGCTTTTTTACCTGCCAGCAGTTCCAGTTGCACTTTGATCTTAATATCGGATGACAT | For constructing *Salmonella* specific suicide plasmids |
| BA4114 | ATGTCATCCGATATTAAGATCAAAGTGCAACTGGAACTGCTGGCAGGTAAAAAAGCTTAA | For constructing *Salmonella* specific suicide plasmids |
| BA4115 | TCATTTGGTCGCGTTATATGCGTTAACCGCATTACCTGCGCCAAAATGTAATGCTTTCAT | For constructing *Salmonella* specific suicide plasmids |
| BA4116 | ATGAAAGCATTACATTTTGGCGCAGGTAATGCGGTTAACGCATATAACGCGACCAAATGA | For constructing *Salmonella* specific suicide plasmids |
| BA4117 | AGTGGATCCCCCGGGCTGCAGGAATTCGATCCGGTATGGGTTCCAGTGCG | For constructing *E. coli* specific suicide plasmids |
| BA4118 | TTATTGCATTGCTTTATAAGCGGTTACCGCATTACCTGCGCCAAAATGTAATGCTTTCAT | For constructing *E. coli* specific suicide plasmids |
| BA4119 | ATGAAAGCATTACATTTTGGCGCAGGTAATGCGGTAACCGCTTATAAAGCAATGCAATAA | For constructing *E. coli* specific suicide plasmids |
| BA4120 | CTCGAGGTCGACGGTATCGATAAGCTTGATGCGGGGTAATACGGAGATACATCATGG | For constructing *E. coli* specific suicide plasmids |
| BA4123 | ATGAAGGTTAATACTATGAAAGCATT | For amplifying *mtlD* from *S.* Typhimurium 14028 |
| BA4124 | CGTCATTTGGTCGCGTTATA | For amplifying *mtlD* from *S.* Typhimurium 14028 |
| BA4127 | AGTGGATCCCCCGGGCTGCAGGAATTCGATCGTAAGTTAAAACAATCAATAGATCCATAA | For constructing *E. coli* specific suicide plasmids |
| BA4128 | TTACTTACGACCTGCCAGCAGTTCCAGCACTTGCACTTTGATCTTAATATCGGATGACAT | For constructing *E. coli* specific suicide plasmids |
| BA4129 | ATGTCATCCGATATTAAGATCAAAGTGCAAGTGCTGGAACTGCTGGCAGG | For constructing *E. coli* specific suicide plasmids |
| BA4130 | GGGTACCGAGCTCGAATTC | pEX18-GM linearization |
| BA4131 | GGGGATCCTCTAGAGTCGAC | pEX18-GM linearization |
| BA4135 | ACCATGATTACGAATTCGAGCTCGGTACCCACGCCAGAAGAGGAAGGAAAGCGACCATTA | For constructing *P. aeruginosa* specific suicide plasmids |
| BA4136 | TCATTCGCCGAGTACCTGGCGCAGGGTTTCCAGGGGGAGGTGCTGCCGGTTGAGTTTCAT | For constructing *P. aeruginosa* specific suicide plasmids |
| BA4137 | ATGAAACTCAACCGGCAGCACCTCCCCCTGGAAACCCTGCGCCAGGTACTCGGCGAATGA | For constructing *P. aeruginosa* specific suicide plasmids |
| BA4138 | ATGCCTGCAGGTCGACTCTAGAGGATCCCCTATTCCGCCATGGCTGATCTCAAGATCCGC | For constructing *P. aeruginosa* specific suicide plasmids |
| BA4143 | CTCGAGGTCGACGGTATCGATAAGCTTGATGCGACGGAAATTGAGATAGCCGATG | For constructing *C. sakazakii* specific suicide plasmids |
| BA4144 | TTACGCCACGGTTTTACCGGAAAGCAGCGCTTGCACTTTGATCTTGATATCGGATGACAT | For constructing *C. sakazakii* specific suicide plasmids |
| BA4145 | ATGTCATCCGATATCAAGATCAAAGTGCAAGCGCTGCTTTCCGGTAAAACCGTGGCGTAA | For constructing *C. sakazakii* specific suicide plasmids |
| BA4146 | TTACGCTGTTGCGTTATACGCGTTTACTGCATTACCTGCGCCAAAATGTAATGCTTTCAT | For constructing *C. sakazakii* specific suicide plasmids |
| BA4147 | ATGAAAGCATTACATTTTGGCGCAGGTAATGCAGTAAACGCGTATAACGCAACAGCGTAA | For constructing *C. sakazakii* specific suicide plasmids |
| BA4148 | AGTGGATCCCCCGGGCTGCAGGAATTCGATTCAGCTTTTCCATATACATATCGGGCGCGT | For constructing *C. sakazakii* specific suicide plasmids |
